# Mechanical Circulatory Support Systems in the Management of Ventricular Arrhythmias: A Contemporary Overview

**DOI:** 10.3390/jcm13061746

**Published:** 2024-03-18

**Authors:** Marco Valerio Mariani, Nicola Pierucci, Pietro Cipollone, Walter Vignaroli, Agostino Piro, Paolo Compagnucci, Andrea Matteucci, Cristina Chimenti, Claudio Pandozi, Antonio Dello Russo, Fabio Miraldi, Carmine Dario Vizza, Carlo Lavalle

**Affiliations:** 1 Department of Cardiovascular, Respiratory, Nephrological, Aenesthesiological and Geriatric Sciences “Sapienza” University of Rome, 00161 Rome, Italy; nicola.pierucci@uniroma1.it (N.P.); pietro.cipollone@uniroma1.it (P.C.); agostino.piro@uniroma1.it (A.P.); cristina.chimenti@uniroma1.it (C.C.); dario.vizza@uniroma1.it (C.D.V.); carlo.lavalle@uniroma1.it (C.L.); 2 Department of Cardiac Surgery, San Carlo di Nancy, GVM Care & Research, 00165 Roma, Italy; vignaroli.walter@gmail.com; 3 Cardiology and Arrhythmology Clinic, Marche University Hospital, 60126 Ancona, Italy; paolocompagnucci1@gmail.com (P.C.); antonio.dellorusso@gmail.com (A.D.R.); 4 Clinical and Rehabilitation Cardiology Division, San Filippo Neri Hospital, 00135 Rome, Italy; andrea.matteucci@aslroma1.it (A.M.); cpandozi@libero.it (C.P.); 5 Cardio Thoracic-Vascular and Organ Transplantation Surgery Department, Policlinico Umberto I Hospital, 00161 Rome, Italy; fabio.miraldi@uniroma1.it

**Keywords:** hemodynamic mechanical support, electrical storm ablation, ECMO

## Abstract

Ventricular tachycardias (VTs) and electrical storms (ES) are life-threatening conditions mostly seen in the setting of structural heart disease (SHD). Traditional management strategies, predominantly centered around pharmacological interventions with antiarrhythmic drugs, have demonstrated limited efficacy in these cases, whereas catheter ablation is related with more favorable outcomes. However, patients with hemodynamically unstable, recurrent VT or ES may present cardiogenic shock (CS) that precludes the procedure, and catheter ablation in patients with SHD portends a multifactorial intrinsic risk of acute hemodynamic decompensation (AHD), that is associated with increased mortality. In this setting, the use of mechanical circulatory support (MCS) systems allow the maintenance of end-organ perfusion and cardiac output, improving coronary flow and myocardial mechanics, and minimizing the effect of cardiac stunning after multiple VT inductions or cardioversion. Although ablation success and VT recurrence are not influenced by hemodynamic support devices, MCS promotes diuresis and reduces the incidence of post-procedural kidney injury. In addition, MCS has a role in post-procedural mortality reduction at long-term follow-up. The current review aims to provide a deep overview of the rationale and modality of MCS in patients with refractory arrhythmias and/or undergoing VT catheter ablation, underlining the importance of patient selection and timing for MCS and summarizing reported clinical experiences in this field.

## 1. Introduction

Ventricular tachycardias (VTs) and electrical storms (ES) are life-threatening conditions mostly seen in the setting of structural heart disease (SHD), (such as in cases of coronary artery disease (CAD), and heart failure with reduced ejection fraction (HFrEF)), with arrhythmias commonly related to re-entry in and around regions of scarring [1,2]. The incidence of VT in this cohort is notably high, presenting a substantial challenge in cardiac care. Within three years postimplantation of an implanted cardioverter-defibrillator (ICD) in primary prevention, between 20% and 35% of HFrEF patients are likely to experience a lifesaving device therapy, whereas 4–7% of patients will -experience ES [3]. Traditional management strategies, predominantly centered around pharmacological interventions with antiarrhythmic drugs, have demonstrated limited efficacy in these cases. In the VANISH trial, catheter ablation proved to be superior to antiarrhythmic drug therapy in terms of the reduction in ES and appropriate ICD therapies, although no differences in mortality were observed [4]. In recent years, mounting evidence has demonstrated the pivotal role of catheter ablation in reducing the arrhythmic burden, improving the prognosis and quality of life of patients with SHD [5,6], as well as in patients with ES [7].

In the context of ventricular arrhythmias, cardiogenic shock (CS), defined as systemic tissue hypoperfusion secondary to impaired cardiac output (CO) despite adequate circulatory volume, and left ventricular filling pressure, may be the result of hemodynamically unstable, recurrent VT/ES or may occur during catheter ablation. Indeed, catheter ablation of VT and ES in patients with SHD portends a multifactorial intrinsic risk of acute hemodynamic decompensation (AHD), defined as sustained hypotension (systolic blood pressure < 90 mmHg) despite increasing doses of vasopressors, requiring emergent placement of mechanical circulatory support (MCS) systems and/or procedure termination. AHD leads to worsened end-organ perfusion, lactic acidosis, and reduced myocardial contractility, eventually resulting in a five-fold increase in post-procedural mortality at follow-up [8]. In the setting of CS secondary to refractory VTs or ES, and in high-risk patients undergoing catheter ablation, MCS improves the mean arterial pressure, maintains end-organ perfusion and allows VT activation and entrainment mapping, thus facilitating the delineation of the VT circuit. The current review aims to provide a deep overview of the rationale and modality of MCS in patients with refractory arrhythmias and/or undergoing VT catheter ablation, underlining the importance of patient selection and timing for MCS, and summarizing reported clinical experiences in this field.

## 2. Rationale for Mechanical Circulatory Support in Patients with Ventricular Arrhythmias

In patients presenting with CS due to refractory VT/ES, hemodynamic stabilization is of paramount importance to avoid end-organ hypo-perfusion and multi-organ failure (MOF), eventually resulting in death. The achievement of hemodynamic stabilization with MCS may be followed by spontaneous restoration of sinus rhythm, but often electric stabilization requires additional treatment using catheter ablation. In CS due to recurrent and refractory VT or ES, MCS allows vital parameter stabilization and HF status optimization before catheter ablation, that would otherwise be pointless in a patient with untreated MOF. In patients presenting for VT ablation procedures, factors contributing to AHD include the severity of heart failure and reduced left ventricular ejection fraction, underlying comorbidities, anesthesia, and the complexity of the VT substrate [9,10]. Patients undergoing VT ablation usually have HFrEF with severe left ventricular systolic dysfunction and lower basal CO. These patients are more likely to experience AHD associated with anesthesia induction, often complicated by hypotension and significant changes in autonomic tone that can predispose them to cardiac ischemia, systemic hypoperfusion and AHD [8,11]. Moreover, recurrent shocks for unstable VT during a procedure may predispose them to a further reduction in cardiac contractility. Interestingly, in patients with SHD and a reduced left ventricular ejection fraction (LVEF), hemodynamic instability may persist even after successful interruption of unstable VT. Skhirtladze at al. showed that the time to recovery of baseline CO after pulseless VT or ventricular fibrillation (VF) defibrillation is related to baseline LVEF, ranging from 0 s in patients with LVEF > 50%, to 17 s in patients < 30% [12]. Moreover, this delayed hemodynamic recovery has been confirmed in a population of 20 patients with an LVEF less than 40% undergoing VT ablation assisted by a pLVAD. In the PERMIT1 study, Miller et al. showed that 17% of unstable VTs led to cerebral hypoperfusion regardless of the presence of a pLVAD, requiring cardioversion [13]. AHD risk is also associated with VT features and mapping strategies. Nowadays, due to the early reperfusion therapy of acute myocardial infarction leading to small dense scar areas and a large border zone with surviving myocardial cells harboring VT circuits, almost 90% of ischemic scar-related VT are fast and unstable in relation to the impaired left ventricular filling time during diastole. In this view, activation and entrainment of VT mapping to delineate the VT circuit portend an increased risk of AHD, resulting in a shift towards substrate mapping strategies during stable sinus or paced rhythm that identify regions of ventricular scar based on tissue electrical voltage, and also conducting channels—regions of slow conduction via fractionated electro grams [14,15]. However, substrate mapping strategies may also be hampered by an increased risk of AHD, mostly related to recurrent VT induction during catheter manipulation, and the need for more extensive mapping and ablation requiring prolonged time under general anesthesia. It is noteworthy that in the series reported by Santangeli et al. [8], AHD occurred during stable sinus or paced rhythm during substrate mapping.

## 3. Devices for Mechanical Circulatory Support

Currently, the available devices for MCS are intra-aortic pump counterpulsation (IABP), the TandemHeart, the Impella, and extracorporeal membrane oxygenation (ECMO). Studies comparing the efficacy of these device are scarce, and the choice of the system depends on the treating physician’s experience, device availability, patient features, and degree of circulatory support needed. Every device has pros and limitations that should guide treatment decisions (Table 1) [8,10,11].

## 4. Intra-Aortic Balloon Pump Counterpulsation

IABPs are the primary temporary mechanical circulatory support systems used, especially in cases of cardiogenic shock or during complex percutaneous procedures. These devices work by placing a balloon in the descending aorta. The placement is critical: the balloon’s far end is positioned just beyond the left subclavian artery’s origin, and the nearer end is above the renal arteries. By inflating during the heart’s relaxation phase (diastole), the balloon boosts diastolic pressure, which in turn enhances blood flow to both the heart and the rest of the body. Additionally, deflating the balloon during the heart’s contraction phase (systole) reduces the workload on the left ventricle, thus improving its performance. However, IABPs have their limitations. They can only increase cardiac output by about 0.5 L/min. For patients with ongoing ventricular tachycardia (VT), the incremental gains in mean arterial pressure and stroke volume that IABPs provide might not meet the body’s hemodynamic needs. The effectiveness of IABPs hinges on the precise timing of balloon inflation and deflation, which should align with either pressure changes or ECG signals. This precise timing demands a heart rhythm that is stable, regular, and not excessively fast (over 120 beats per minute), making IABPs less suitable for patients undergoing VT ablation. The triggers for inflating the balloon during VT should be either a peak in the QRS complex on a surface ECG, or a cue from the arterial pressure waveform [16]. Despite these constraints, IABPs are favoured for several reasons: they require relatively small arterial sheaths (7.5 Fr to 8 Fr), are easy to insert, and are well-known to lab staff.

## 5. TandemHeart

The TandemHeart, developed by CardiacAssist Inc, is a percutaneous system that creates a bypass from the left atrium to the femoral artery. It uses an external centrifugal pump capable of delivering a flow rate between 3.5 and 5 L/min [16]. 

To implant this device, initial venous access is established. This is followed by a transseptal puncture and subsequent dilation to fit a 21 Fr inflow cannula into the left atrium. The correct placement of the transseptal cannula is verified using fluoroscopy, where dye is injected to ensure all side ports of the cannula have passed through the interatrial septum. Alternatively, intracardiac echocardiography may help in verifying the correct position of the system. Additionally, a femoral artery angiogram is performed to confirm the puncture site is appropriately positioned above the common femoral artery bifurcation, and to check for any significant peripheral arterial disease. The site is then prepared by dilating the arteriotomy with sheaths ranging from 6 Fr to 8.5 Fr and eventually 15 Fr to 17 Fr for the arterial perfusion cannula. Before moving to the larger sheath, the arteriotomy site is often pre-closed using two orthogonally placed 6 Fr Perclose vascular closure devices from Abbott Laboratories [17]. Once set up, the transseptal and arterial cannulae are connected to their respective ports on the external pump. Heparin is administered initially as a bolus before the transseptal puncture, followed by an infusion to maintain an activated clotting time (ACT) of at least 300 s. The device’s speed is typically set between 3000 to 7500 rpm and adjusted to provide the necessary hemodynamic support. A significant limitation of this percutaneous left ventricular assist device (pLVAD) is the requirement for large venous and arterial access points, elevating the risk of vascular complications. The size of the transseptal venous cannula may interfere with transseptal mapping of the left ventricle, often necessitating a retrograde aortic approach for mapping and ablation. This approach can adversely affect the accuracy of mapping and the contact force during ablation. Potential complications of the TandemHeart system include cardiac tamponade, bleeding, critical limb ischemia, sepsis, arrhythmias, and residual atrial septal defects [16].

## 6. Impella

The Impella device, produced by Abiomed, represents a significant advancement in pLVAD technology. It features a compact, impeller-driven axial flow pump that is temporarily inserted across the aortic valve. This device functions by actively pumping blood from the left ventricle directly into the ascending aorta. The Impella has been primarily utilized in patients undergoing high-risk percutaneous coronary interventions, and those suffering from cardiogenic shock. In these cases, the Impella 2.5 model has demonstrated superior capabilities in enhancing cardiac index and mean arterial pressure compared to IABP [18]. Three distinct versions of the Impella are particularly relevant for hemodynamic support during VT ablation: (1) the Impella 2.5, which is inserted via a 13 Fr introducer sheath in the femoral artery and can deliver up to 2.5 L/m; (2) the Impella CP, which requires a 14 Fr access sheath and provides around 3.5 L/m; and (3) the Impella 5.0, necessitating surgical arterial access (21 Fr sheath) and capable of offering up to 5.0 L/m. Among these, the Impella 2.5 has seen the most clinical use in VT ablation procedures. The implantation technique involves first obtaining percutaneous vascular access in the common femoral artery, typically on the left side, ensuring the access point is above the bifurcation of the common femoral artery. A contrast agent is used to confirm the access level and to check for significant peripheral arterial disease. The arteriotomy tract is then prepared using a sheath ranging from 6 Fr to 8.5 Fr in diameter. Before installing the larger 13 Fr sheath, the arteriotomy site is pre-closed using two 6 Fr Perclose vascular closure devices, placed orthogonally [16]. This preclosure technique is crucial for rapid hemostasis following the removal of the arterial sheath at the procedure’s conclusion. Once the sheath is upsized to the final size necessary for the pLVAD system, anticoagulation is initiated with intravenous heparin to achieve a target activated clotting time (ACT) of over 250 s. Maintaining an ACT above 250 s before removing the dilator is important to prevent thrombus formation that could obstruct the catheter. The pLVAD is then carefully advanced in a retrograde manner through the aorta over a 0.018-inch guidewire and positioned across the aortic valve. The device’s inlet is situated in the left ventricle, approximately 4 cm below the aortic valve annulus, with the outlet in the aortic root. The correct positioning is confirmed using fluoroscopy and intracardiac echocardiography. Once in place, the device is activated and gradually adjusted to its full support capability. After installing the Impella device, it is crucial to frequently recheck its positioning using the waveform displayed on the console. This is because the device’s position might shift during VT episodes or when tachycardia suddenly stops. If displacement occurs, it can usually be rectified by slightly adjusting the device’s shaft at the femoral access point. The timing of anticoagulant administration is influenced by whether epicardial mapping and ablation are planned. If these procedures are the primary focus, pericardial access is usually established at the procedure’s beginning. In such cases, the placement of the Impella is delayed until it is certain that there is no pericardial bleeding. Alternatively, if pericardial access is sought after the Impella’s placement, the device may be temporarily withdrawn from the left ventricle and anticoagulation reversed to facilitate access. Once pericardial bleeding is ruled out, anticoagulation can be resumed and the Impella repositioned. If the device is not completely removed during this process, it should at least be retracted into the descending aorta, keeping a low performance level and continuing the purge solution to prevent clot formation.

Electromagnetic interference (EMI) with the Impella can occur when using magnetic-based electroanatomic mapping systems, such as CARTO. This interference might manifest as temporary mapping disruptions or distortions in catheter positioning. EMI, often mild, is more pronounced when mapping near the ventricular outflow tract or the epicardial anterior base, due to the proximity of the device’s motor. To mitigate EMI, it might be necessary to lower the Impella’s performance level. However, most EMI cases do not require significant intervention. When using the Impella, a transseptal approach for left-sided ablation is preferred due to the reduced EMI compared to a retrograde approach. The Impella CP model seems to produce less EMI, possibly due to better motor insulation. Impella devices offer advantages over the TandemHeart, such as the more efficient reduction of left ventricular end-diastolic pressure and myocardial oxygen demand at comparable flow rates [16]. They also require smaller arterial sheaths and eliminate the need for additional venous access and transseptal puncture, which could reduce vascular complication risks and shorten implantation times. However, complications like vascular injuries; hematomas; pseudoaneurysms; retroperitoneal bleeding; aortic valve damage; stroke; systemic embolism; arrhythmias; catheter manipulation difficulties; and thrombosis are associated with Impella placement. Vascular complications are less common with the smaller Impella 2.5 compared to the larger Impella 5.0. In severe heart failure or cardiogenic shock cases, the greater hemodynamic support provided by the Impella 5.0 or TandemHeart may be necessary, despite their higher complication rates and longer implantation times [16].

## 7. ECMO

The veno-arterial ECMO (VA-ECMO) uses a centrifugal pump that allows the collection of deoxygenated blood from the venous system, via a 19- to 25 Fr cannula positioned in the right atrium via femoral vein, to an external membrane oxygenator system that provides gas exchange. The oxygenated blood is then pumped into the arterial system from a 17 to 21 Fr cannula positioned in the aorta via the femoral artery [19]. VA-ECMO is the only HMS capable of providing complete biventricular support and is the device of choice in patients with severe right ventricular (RV) dysfunction, whereas Impella and TandemHeart overload RV, possibly leading to RV failure in cases of pre-existent RV disease. VA-ECMO is the most effective MCS in patients with recurrent VT or ES, supplying end-organ perfusion in patients with refractory cardiopulmonary impairment. Both venous and arterial cannulas may be positioned percutaneously, or the vascular accesses may be gained surgically [20]. The system requires systemic anticoagulation with a target ACT > 250 s. Of note, VA-ECMO increases LV afterload, and consequently wall stress and oxygen demands. To counteract this secondary effect, different LV venting strategies have been developed, including reduction of ECMO flow, ultrafiltration or hemodialysis, inotropes, IABP, or Impella to reduce LV afterload [21].

## 8. MCS and Ventricular Arrhythmia Ablation: Patient Selection 

AHD during VT ablation procedures is associated with worse prognosis, hence the identification of patients at high-risk of periprocedural AHD is of paramount importance. On the one hand MCS may increase the safety profile of catheter ablation, on the other hand MCS should be proposed only to the high-risk subgroup of patients to reduce the costs and HMS-related complications. In this view, the pre-procedural identification of patients at high-risk of AHD may allow pre-procedural optimization of HF status and adequate procedural planning, including MCS implantation. Risk stratification tools have been evaluated to assess the risk of AHD in patients undergoing VT ablation and to guide the selection of patients for MCS. In a series of 193 consecutive patients undergoing VT ablation, Santangeli et al. [8] found an AHD rate of 11%. Using independent variables significantly associated with AHD at univariate regression analysis, they found 7 variables that were incorporated in a score, known as the PAINESD risk score, which considers as predictors of acute hemodynamic collapse advanced age; ischemic cardiomyopathy; severe left ventricular (LV) dysfunction and heart failure; presentation during an electrical storm; and comorbidities like diabetes and obstructive pulmonary disease (Table 2). The PAINESD score allowed the division of the population into three groups, each with a different risk of AHD: a low risk group (PAINESD ≤ 8) with an AHD risk of 1%; an intermediate risk group (PAINESD ranging from 9 to 14) with an AHD risk of 6%; and a high-risk group (PAINESD ≥ 15) with a 25% risk of AHD. In this series, AHD was associated with a 50% mortality as compared with 11% in patients without AHD. The PAINESD score was tested in an independent cohort of 93 patients undergoing scar-related VT ablation [22]. Patients experiencing AHD and subsequent rescue pLVAD insertion had significantly higher PAINESD scores than patients without AHD (17.8 ± 3.8 vs. 13.4 ± 5.4, respectively, *p*-value 0.01). In a large retrospective multicenter series of 2061 patients with SHD undergoing VT ablation, the PAINESD score was significantly higher in patients who died during a 30-day follow-up and in patients requiring urgent pLVAD insertion due to AHD [23]. In 2018, Muser et al. [24] demonstrated that the PAINESD score is a reliable tool to guide pre-emptive pLVAD insertion in a propensity-matched cohort of patients undergoing scar-related VT ablation: a significant mortality benefit (risk reduction of 57%) was only observed in patients at high-risk of AHD as stratified by the score (PAINESD ≥ 15), whereas a non-significant effect was found in patients at low-risk of AHD (PAINESD ≤ 8). Recently, John et al. [25] evaluated the effect of ventricular scar burden, (known as total scar volume (TSV), determined via a pre-procedural computed tomography scan), on the risk of periprocedural AHD. In this retrospective study, among 61 patients with TSV data, AHD occurred in 31 cases (21%) and TSV was strongly associated with AHD. The addition of TSV into the PAINESD risk score, resulted in the revised score named PAINES2D, that proved to be more accurate in identifying patients at high risk of periprocedural AHD. Indeed, the PAINES2D score predicted AHD development with higher accuracy as compared with the PAINESD score (area under the curve: 0.73, *p*-value 0.011 and area under the curve: 0.67, *p*-value 0.058). 

Vergara et al. evaluated potential factors associated with mortality and VT recurrence in 1251 patients undergoing VT ablation, using survival tree analysis methods [26]. Consistent with the PAINESD score, they found that LVEF, ES, and previous ablation were the best predictors of mortality, whereas LVEF, an ICD/cardiac resynchronization device, and previous ablation best predicted VT recurrence. The I-VT score allowed patients to be allocated to three different risk groups on the basis of survival analysis, with 1-year mortality ranging from 35% in the high-risk group to 2.8% in the low-risk group. Interestingly, as compared with the PAINESD score, I-VT score provides a 1-year outcome probability estimation and shows a larger area under the curve when predicting the risk of mortality after VT ablation [26].

## 9. MCS and Ventricular Arrhythmias Ablation: Implantation Timing

MCS is often employed as a last-minute “rescue” measure during VT ablation in patients who experience hemodynamic collapse and CS that do not respond to vasopressors and inotropes. However, as shown by Santangeli et al., the use of MCS after AHD has a negative prognostic impact with mortality during 30-day follow-up as high as 50% [8]. Enriquez et al. described the outcomes of a cohort of 21 patients undergoing ES ablation, for whom rescue ECMO support was required for AHD during the procedure. Although acute procedural success was achieved in 83% of cases, 88% died after a follow-up of 10 days, mostly for refractory HF [27]. Conversely, Baratto et al. reported more favourable outcomes in a cohort of 64 high-risk patients who had undergone unstable VT ablation using pre-emptive ECMO (in 59 out 64 patients, 92%), whereas in the last 5 patients rescue ECMO was used after AHD development. There was only one in-hospital death (1.5%) due to acute HF and after a mean follow-up of 23 months overall survival was 88%, with 67% of patients free from VT recurrence [28]. In 2017, Mathuria et al. compared the 30-day mortality rate among high-risk patients undergoing VT ablation with pre-emptive insertion of pLVAD, with patients who received rescue pLVAD after AHD development. Patients with pre-emptive pLVAD had a significantly lower mortality than patients with urgent MCS device insertion after AHD (4% vs. 58.3%, *p*-value 0.003), whereas no difference was found in mortality among high-risk patients receiving pre-emptive pLVAD and low-risk patients in whom the mechanical support was not used [22]. In a propensity-matched cohort of 150 patients undergoing scar-related VT ablation, Muser et al. [24] demonstrated that the prophylactic use of Impella was associated with a significantly lower risk of AHD, as compared to not use HMS at all (7% vs. 23%, *p* -value 0.03).

These findings suggest that MCS should be used pre-procedurally to exert beneficial effect and not as a rescue strategy. Hence, a comprehensive pre-procedural patient assessment to identify high-risk features for AHD should be prioritized. The use of tools such as the PAINESD score and I-VT score have demonstrated good accuracy in predicting AHD risk and should be part of pre-procedural evaluation of every patient.

## 10. Efficacy and Safety of MCS in VT Ablation

Data evaluating the role of MCS during VT ablation procedures come from observational studies, due to the lack of prospective randomized controlled trials in this field. However, some important insights may be derived from the analysis of these studies (Table 3). Different studies did not report any advantage in terms of VT recurrence, heart transplantation, and mortality between the group with pLVAD and the one without MCS, although pLVAD insertion allowed the induction and ablation of a significantly higher number of VTs without increasing the AHD rate [29,30]. The lack of benefit of MCS on VT ablation outcomes has been confirmed by a recent meta-analysis of five observational studies by Mariani et al. [31], totalling 394 patients undergoing 400 procedures for unstable VT, and comparing the outcomes associated with the use of prophylactic pLAVD vs. no temporary MCS. Of note, 55% of the included patients had ischemic cardiomyopathy, and in the pLVAD group 86.6% received Impella, whereas the last 13.4% received TandemHeart. The use of pre-emptive pLAVD resulted in numerically more VT being induced and longer mapping time in VT, without any difference in procedural success and VT recurrences among the groups [31]. Moreover, Muser et al. [24] did not find a significant difference in the cumulative incidence of VT recurrence comparing the use of upfront pLVAD vs. no pLVAD. As suggested by Virk et al. [19], the disappointing results of MCS use on ablation outcomes may be explained by the heterogeneity of substrates in the available studies. Indeed, as compared with ischemic cardiomyopathy, VT ablation in non-ischemic cardiomyopathy (NICM) is more likely to require longer mapping time and activation mapping, due to the lack of significant substrate and an ablation target when only using substrate mapping. Hence, in this subgroup of patients the use of MCS may provide the best impact on ablation outcomes. In this regard, Aryana et al. [32] demonstrated that the use of Impella in patients with NICM was associated with a significant reduction in recurrent ICD therapies and redo-ablation. When considering other outcomes related to MCS use, different papers have provided encouraging evidence. Muser et al. [24] showed a lower rate of death or heart transplant at 12 months follow-up (33% vs. 66%, *p*-value < 0.01) with the use of pre-emptive pLVAD. In the same vein, in a Medicare claim analysis of 345 patients who had undergone VT ablation, Aryana et al. [33] found that pLVAD as compared with IABP significantly reduced in-hospital mortality (6.5% vs. 19.1%), 30-days rehospitalisation (27% vs. 38.7%), and in-hospital renal failure (11.7% vs. 21.7%). Kuo et al. [34], in a retrospective study of 317 patients, showed that AHD was associated with a 4-fold increase in the risk of acute kidney injury (AKI), which in turn predicted 1-year mortality. The meta-analysis by Mariani et al. [31] confirmed these results, pointing toward an improved survival when using pre-emptive MCS in VT ablation. Indeed, the analysis showed a non-significant trend towards in-hospital/30-day mortality reduction with upfront pLVAD use (OR 0.55, 95% CI 0.28–1.05, *p*-value 0.07), and a significant reduction in mortality at 3–19 months of follow-up (OR 0.55, 95% CI 0.32–0.94, *p*-value 0.03). The main effect of MCS is the maintenance of end-organ perfusion and CO, improving coronary flow and myocardial mechanics, and minimizing the effect of cardiac stunning after multiple VT inductions or cardioversion. Although ablation success and VT recurrence are not influenced by hemodynamic support devices, MCS promotes diuresis and reduces the incidence of post-procedural AKI. In addition, MCS may have a role in mortality reduction at long-term follow-up. This evidence emphasizes the importance of using these devices and highlights the need for more substantial evidence to firmly establish their role in improving patient outcomes after VT ablation. 

A systematic review by Mariani et al. [40] underlined differences among MCS systems in terms of levels and characteristics of support. Reddy et al. [39] showed the superiority of Impella and TandemHeart compared to IABP. Similarly, Aryana et al. showed better outcomes related with the use of pLVAD as compared with IABP [33]. As previously stated, although IABP is easily inserted and managed, with fewer complications, its reduced benefit is likely related to the modest increase in CO and susceptibility to asynchronous counterpulsation in the context of fast VT. When comparing VA-ECMO, Impella 2.5, and TandemHeart, Ostadal et al. showed that for a ventricular pacing rate > 300 beat/min and during sustained VF, ECMO was the only MCS capable of maintaining mean arterial pressure > 70–80 mmHg, with Impella 2.5 showing the lowest efficacy [41]. Hence, MCS devices seem to have different levels of support capabilities, although randomized controlled trials are needed to draw solid conclusions.

Regarding safety, cardiac tamponade has been reported using pLVAD with a rate ranging from 3–11%, whereas an analysis of 230 patients receiving pLVAD reported a vascular complication rate as low as 1.7% [39]. However, other series showed an access-site complications rate as high as 16%, with the most common complication represented by local hematoma or bleeding [13]. No studies reported an increased incidence of stroke or systemic embolism with the use of either TandemHeart or Impella.

## 11. Multidisciplinary Approach for VT Management

Patients undergoing VT ablation often have significant comorbidities, such as diabetes; HF; ischemic heart disease; atrial fibrillation; chronic ischemic leukoencephalopathy; and advanced-stage renal failure [42]. Treating such compromised patients can be challenging, thus a multidisciplinary approach involving various professionals could improve the standard of care for patients undergoing VT ablation under deep sedation and with MCS. 

A multidisciplinary approach in patient management involves the participation of multiple professionals in the care of the patient. In the case of VT ablations, the team should consist of electrophysiologists for the study and treatment of the arrhythmic event to be corrected; a cardio-anesthesiologist for patient management during deep sedation; a perfusion technician for the management of MCS; and an intensive care cardiologist for postoperative patient management [43]. Each professional should care for and manage a specific moment in the treatment of the complicated patient. The team should collaboratively analyze each case to organize every phase of patient care. Of special importance are the pre-operative prophylaxis for patients with prosthetic valves, allergies, or intolerances to drugs essential for the procedure; the study phase in the operating room, (perhaps the most delicate moment), during which the interventional electrophysiologist, cardio-anesthesiologist, and perfusion technician must work together to ensure proper perfusion of the vital and often already compromised organs (such as the brain, kidneys, heart, and liver), provide appropriate sedation to the patient, and eliminate the cause of the arrhythmia; and finally, managing the patient during the post-operative phase in intensive care is of crucial importance to ensure a quick and complete recovery for the patient who has just undergone often invasive and complicated medical procedures [43]. Della Bella et al. [44] reported the management of VT in the setting of a dedicated VT unit based on a multidisciplinary approach. They found that catheter ablation in the context of a multidisciplinary model, aiming for the comprehensive management of VT patients, prevents long-term VT recurrences and reduces mortality, especially in patients with non-inducibility of any VT at programmed ventricular stimulation after the ablation procedure. More recently, Pothineni et al. [45] presented the results of a pilot study in which a multidisciplinary management pathway for eight patients with advanced HF and ES with high PAINESD scores (>17) was developed and implemented. The multidisciplinary approach involved the collaboration between cardiac electrophysiologists; advanced HF specialists; and cardiothoracic surgeons, and included pre-procedural hemodynamic optimization; evaluation for advanced HF therapy options; and prophylactic initiation of VA-ECMO. The authors reported a good short and long-term mortality, as well as good VT control, associated with the multidisciplinary approach. Acute clinical success with elimination of the clinical VT was achieved in all patients, without clinical VT recurrence during in-hospital stay. One patient died of refractory shock and inability to wean from ECMO. During a mean follow-up of 14 ± 16 months, two patients had VT recurrence and none of the patients underwent LVAD/transplant.

## 12. Conclusions

MCS devices are effective in the treatment of CS related to refractory VT or ES, and have shown effectiveness in reducing AHD and its consequences in patients undergoing VT ablation. Risk stratification for AHD should be calculated using tools such as the PAINES2D score, and should guide the optimization of procedural planning, leading to pre-emptive device implantation in high-risk patients. MCS use is associated with reduced mortality at follow-up, probably due to the maintenance of end-organ perfusion and cardiac contractility during the ablation procedure. Larger, randomized studies are awaited to fill the current gaps in knowledge in this field, and to provide stronger evidence of the role of MCS devices in improving patient and procedural outcomes after VT ablation.

## Figures and Tables

**Table 1:** Characteristics of the main mechanical support systems.

Device	Method of Insertion	Support Mechanism	CO Increase	Benefits	Drawbacks	When Not to Use (Contraindications)	Main Risks
**IABP**	Via skin or surgically, using 7.5–8F size	Counterpulsation (reduces systolic load, boosts diastolic)	0.5 L/min	Known technology, easy to place, small vascular entry	Limited CO boost, relies on ECG/pressure triggers, suboptimal for VT patients	Moderate/severe aortic insufficiency, aortic pathology, serious peripheral artery disease	Limb blood flow issues, vascular harm, brain stroke
**TandemHeart**	Skin or surgical approach, 21F venous and 15/17F arterial	Centrifugal pump providing constant flow	3.5–5.0 L/min	Supports part of left ventricle function	Needs bigger vascular cannulas, requires puncturing the interatrial septum, requires a retrograde transaortic approach to LV mapping	Serious peripheral artery disease, right ventricle failure	Limb blood flow issues, vascular harm, heart compression, brain stroke, remaining atrial septal hole, bleeding
**Impella 2.5**	Either through skin or surgery, with a 13F arterial access	Axial pump moving blood from left ventricle to aorta	2.5 L/min	Assists part of left ventricle	Requires big arterial access, possible EMI during VT mapping	Mechanical aortic valve, narrow aortic opening, significant aortic insufficiency, left ventricle clot, serious peripheral artery disease, hole in the heart wall, right ventricle failure	Limb blood flow issues, vascular harm, perforation, brain stroke
**Impella CP**	Inserted through skin or surgery, with a 14F arterial access	Same as Impella 2.5, but with increased flow	3.5 L/min	Supports part of left ventricle	Needs larger arterial access, possible EMI during VT mapping	(Similar to Impella 2.5)	Limb blood flow issues, vascular harm, perforation, brain stroke
**Impella 5.0**	Surgical insertion (femoral/axillary), with a 21F access	Similar to other Impellas, with maximum support	5 L/min	Full support for left ventricle	Requires the largest arterial access, possible EMI during VT mapping	(Similar to Impella 2.5)	Limb blood flow issues (highest risk), vascular harm, perforation, brain stroke
**VA-ECMO**	Through skin or surgery, using 17–22F venous and 15F arterial cannulas	Centrifugal pump with an advanced oxygenator	>4.5 L/min	Top-tier cardiopulmonary support, useful in severe right ventricle failure	Bigger vascular cannulas, complex setup, need for perfusionist	Serious peripheral artery disease, uncontrolled bleeding disorders	Limb blood flow issues, vascular harm, bleeding, infection, blood clots in the system

CO: cardiac output; EMI: electromagnetic interference; IABP: intra-aortic balloon pump counterpulsation; LV: left ventricle; VT: ventricular tachycardia.

**Table 2:** Clinical variables associated with acute hemodynamic decompensation included in the PAINESD Score.

Variable	Score
COPD	5
Age > 60 years	3
Ischemic Cardiomyopathy	6
NYHA Class III or IV	6
LVEF < 25%	3
Storm VT	5
Diabetes Mellitus	3

COPD: chronic obstructive pulmonary disease; LVEF: left ventricular ejection fraction; NYHA: New York Heart Association; VT: ventricular tachycardia.

**Table 3:** Studies of percutaneous hemodynamic support for ventricular tachycardia ablation.

Study	Treatment Group	Control Group	Age, years	LVFE	Acute Success, %	Haemodynamic Support	Recurrence of VT (%)	Mortality/ Transplant, %	Follow-Up
Carbucicchio et al. 2009 [35]	19	NO control group	61 ± 6	NA	68	CPS	50	21	Mean: 42 months
Abuissa et al. 2010 [36]	3	NO control group	55 (mean)	NA	100	Impella			Mean: 7 months
Miller et al. 2011 [37]	10	IABP: 6 NO MHS: 7	15	31 ± 16	PLVAD group: 75 control group: 67	Impella	PLVAD group: 30 control group: 31		3 months
Lu et al. 2013 [38]	16	No control group	63± 11	20 ± 9	ECMO 60 Impella: 60 LVAD: SO	ECMO: 5 patients Impella: 5 patients LVAD: 6 patients	50	6	3 months
Miller et al. 2013 [13]		Patients used as own controls	59 ± 12	30 ± 7	50	Impella 2.5	20	10	1 months
Aryana et al. 2014 [32]	68	34	12	32 ± 10	PLVAD group: 7.1 Control group: 71	Impella 2.5/Impella CP	PLVAD group: 26 Control group: a 41	pLVAD group: O control group: 6	19 ± 12 months
Reddy et al. 2014 [39]		ABP: 22	PLVAD group: 66 ± 12, Control 69 ± 10	pLVAD 29 ± 15 Control 25 ± 10	PLVAD group: 89 control group: 86	Impella 2.5 TandemHeart 19	PLVAD group: 42 control group: 50	PLVAD group: 36 control group: 36	12 ± 5 month
Baratto 2016 [28]	64	No control group	15	27 ± 9	69	ECMO	33	12	Median: 21 months
Aryana al. 2017 [33]		ABP: 115		NA		PLVAD not otherwise specified	PLVAD group: 10.2 Control group: 14.0	pLVAD group: 6.5 Control group: 19.1	12 months
Enriquez et al. [27]	21	No control group	60 ± 11	21 ± 13	83	ECMO (rescue due to AHD)	30	76	Median: 10 days
Kusa et al. 2017 [29]	109	−85	PLVAD group: 64 ± 11 Control group: 61 ± 15	pLVAD: 26 ± 10 Control: 39 ± 16	pLVAD group: 80 Control group: 93	80 with Impella 2.5 and 29 With impella CP	PLVAD group: 32 control group: 21	pLVAD: 12 control: 6	Median: 215 days
Mathuria et al. 2017 [22]	Rescue pLVAD: 12 Pre-emptive PLVAD: 24		Pre-emptive PLVAD group: 65.8 ± 14 control group: 64.8 ± 29 rescue PLVAD: 68.8 ± 8	Pre-emptive PLVAD group: 26 ± 9 control group:28 ± 5 rescue PLVAD: 24 ± 14	Pre-emptive PLVAD group: 61 control group: 66 rescue PLVAD: 50	Impella/ TandemHeart	Pre-emptive PLVAD group: 4 control group: 3.5 Rescue PLVAD: 58.3	pre-emptive pLVAD group: 26 Control group: 44 Rescue PLVAD: 40	3 months
Muser et al. 2018 [24]	Pre-emptive pLVAD: 75	75	PLVAD group: 65 ± 12 Control group: 64 ± 14	PLVAD group: 27 ± 10 Control group: 27 ± 12	PLVAO group: 81 Control group: 62	Impella 2.5/Impella CP	PLVAD group: 33 Control group: 66	PLVAD group: 81 Control group: 62 Control group: 41	12 months
John et al. 2023 [25]	None: 55 Support device (not se 6)	No control group	Median 69	35 ± 14	43	None			12 months

CPS: cardiopulmonary support; ECMO: extracorporeal membrane oxygenation; IABP: intra-aortic balloon counterpulsation; pLVAD: percutaneous left ventricular assist device; VT: ventricular tachycardia. Inizio modulo.

## Data Availability

No new data were created or analyzed in this study. Data sharing is not applicable to this article.

## References

[1] Sirichand S., Killu A.M., Padmanabhan D., Hodge D.O., Chamberlain A.M., Brady P.A., Kapa S., Noseworthy P.A., Packer D.L., Munger T.M. (2017). Incidence of idiopathic ventricular ar-rhythmias: A population-based study. Circ. Arrhythmia Electrophysiol..

[2] Pandozi C., Mariani M.V., Chimenti C., Maestrini V., Filomena D., Magnocavallo M., Straito M., Piro A., Russo M., Galeazzi M. (2022). The scar: The wind in the perfect storm—Insights into the mysterious living tissue originating ventricular arrhythmias. J. Interv. Card. Electrophysiol..

[3] Guarracini F., Casella M., Muser D., Barbato G., Notarstefano P., Sgarito G., Marini M., Grandinetti G., Mariani M.V., Boriani G. (2020). Clinical management of electrical storm: A current overview. J. Cardiovasc. Med..

[4] Sapp J.L., Wells G.A., Parkash R., Stevenson W.G., Blier L., Sarrazin J.F., Thibault B., Rivard L., Gula L., Leong-Sit P. (2016). Ventricular Tachycardia Ablation versus Escalation of Antiarrhythmic Drugs. N. Engl. J. Med..

[5] Santangeli P., Muser D., Maeda S., Filtz A., Zado E.S., Frankel D.S., Dixit S., Epstein A.E., Callans D.J., Marchlinski F.E. (2016). Comparative effectiveness of antiarrhythmic drugs and catheter ablation for the prevention of recurrent ventricular tachycardia in patients with implantable cardioverter-defibrillators: A systematic review and meta-analysis of randomized controlled trials. Heart Rhythm.

[6] Kumar S., Romero J., Mehta N.K., Fujii A., Kapur S., Baldinger S.H., Barbhaiya C.R., Koplan B.A., John R.M., Epstein L.M. (2016). Long-term outcomes after catheter ablation of ventricular tachycardia in patients with and without structural heart disease. Heart Rhythm.

[7] Nayyar S., Ganesan A.N., Brooks A.G., Sullivan T., Roberts-Thomson K.C., Sanders P. (2012). Venturing into ventricular arrhythmia storm: A systematic review and meta-analysis. Eur. Heart J..

[8] Santangeli P., Muser D., Zado E.S., Magnani S., Khetpal S., Hutchinson M.D., Supple G., Frankel D.S., Garcia F.C., Bala R. (2015). Acute hemodynamic decompensation during catheter ablation of scar-related ventricular tachycardia: Incidence, predictors, and impact on mortality. Circ. Arrhythmia Electrophysiol..

[9] Sauer W.H., Zado E., Gerstenfeld E.P., Marchlinski F.E., Callans D.J. (2010). Incidence and predictors of mortality following ablation of ventricular tachycardia in patients with an implantable cardioverter-defibrillator. Heart Rhythm.

[10] Neuzner J., Dietze T., Paliege R., Gradaus R. (2019). Effectiveness of a percutaneous left ventricular assist device in preventing acute hemodynamic decompensation during catheter ablation of ventricular tachycardia in advanced heart failure patients: A ret-rospective single-center analysis. J. Cardiovasc. Electrophysiol..

[11] Reich D.L., Hossain S., Krol M., Baez B., Patel P., Bernstein A., Bodian C.A. (2005). Predictors of Hypotension After Induction of General Anesthesia. Obstet. Anesth. Dig..

[12] Skhirtladze K., Mora B., Moritz A., Birkenberg B., Ankersmit H.J., Dworschak M. (2010). Impaired recovery of cardiac output and mean arterial pressure after successful defibrillation in patients with low left ventricular ejection fraction. Resuscitation.

[13] Miller M.A., Dukkipati S.R., Chinitz J.S., Koruth J.S., Mittnacht A.J., Napolitano C., d’Avila A., Reddy V.Y. (2013). Percutaneous hemodynamic support with Impella 2.5 during scar-related ventricular tachycardia ablation (PERMIT 1). Circ. Arrhythmia Electrophysiol..

[14] Lavalle C., Mariani M.V., Piro A., Straito M., Severino P., Della Rocca D.G., Forleo G.B., Romero J., Di Biase L., Fedele F. (2019). Electrocardiographic features, mapping and ablation of idiopathic outflow tract ventricular arrhythmias. J. Interv. Card. Electrophysiol..

[15] Muser D., Tritto M., Mariani M.V., Di Monaco A., Compagnucci P., Accogli M., De Ponti R., Guarracini F. (2021). Diagnosis and Treatment of Idiopathic Premature Ventricular Contractions: A Stepwise Approach Based on the Site of Origin. Diagnostics.

[16] Naidu S.S. (2011). Novel percutaneous cardiac assist devices: The science of and indications for hemodynamic support. Circulation.

[17] Mahadevan V.S., Jimeno S., Benson L.N., McLaughlin P.R., Horlick E.M. (2008). Pre-closure of femoral venous access sites used for large-sized sheath insertion with the Perclose device in adults undergoing cardiac intervention. Heart.

[18] Seyfarth M., Sibbing D., Bauer I., Fröhlich G., Bott-Flügel L., Byrne R., Dirschinger J., Kastrati A., Schömig A. (2008). A Randomized Clinical Trial to Evaluate the Safety and Efficacy of a Percutaneous Left Ventricular Assist Device Versus Intra-Aortic Balloon Pumping for Treatment of Cardiogenic Shock Caused by Myocardial Infarction. J. Am. Coll. Cardiol..

[19] Virk S.A., Keren A., John R.M., Santageli P., Eslick A., Kumar S. (2019). Mechanical Circulatory Support During Catheter Ablation of Ven-tricular Tachycardia: Indications and Options. Heart Lung Circ..

[20] Della Bella P., Radinovic A., Limite L.R., Baratto F. (2021). Mechanical circulatory support in the management of life-threatening ar-rhythmia. Europace.

[21] Guglin M., Zucker M.J., Bazan V.M., Bozkurt B., El Banayosy A., Estep J.D., Gurley J., Nelson K., Malyala R., Panjrath G.S. (2019). Venoarterial ECMO for Adults: JACC Scientific Expert Panel. J. Am. Coll Cardiol..

[22] Mathuria N., Wu G., Rojas-Delgado F., Shuraih M., Razavi M., Civitello A., Simpson L., Silva G., Wang S., Elayda M. (2017). Outcomes of pre-emptive and rescue use of per-cutaneous left ventricular assist device in patients with structural heart disease undergoing catheter ablation of ventricular tachycardia. J. Interv. Card. Electrophysiol..

[23] Santangeli P., Frankel D.S., Tung R., Vaseghi M., Sauer W.H., Tzou W.S., Mathuria N., Nakahara S., Dickfeldt T.M., Lakkireddy D. (2017). Early Mortality After Catheter Ablation of Ventricular Tachycardia in Patients with Structural Heart Disease. J. Am. Coll. Cardiol..

[24] Muser D., Liang J.J., Castro S.A., Hayashi T., Enriquez A., Troutman G.S., McNaughton N.W., Supple G., Birati E.Y., Schaller R. (2018). Outcomes with prophylactic use of percutaneous left ventricular assist devices in high-risk patients undergoing catheter ablation of scar-related ventricular tachycardia: A propen-sity-score matched analysis. Heart Rhythm.

[25] John L.A., John I.I., Tedford R.J., Gregoski M.J., Gold M.R., Field M.E., Payne J.E., Schoepf U.J., Suranyi P., Cochet H. (2023). Substrate Imaging Before Catheter Ablation of Ventricular Tachycardia: Risk Prediction for Acute Hemodynamic Decompensation. JACC Clin. Electrophysiol..

[26] Vergara P., Tzou W.S., Tung R., Brombin C., Nonis A., Vaseghi M., Frankel D.S., Di Biase L., Tedrow U., Mathuria N. (2018). Predictive Score for Identifying Survival and Recurrence Risk Profiles in Patients Undergoing Ventricular Tachycardia Ablation: The I-VT Score. Circ. Arrhythmia Electrophysiol..

[27] Enriquez A., Liang J., Gentile J., Schaller R.D., Supple G.E., Frankel D.S., Garcia F.C., Wald J., Birati E.Y., Rame J.E. (2018). Outcomes of rescue cardiopulmonary support for periprocedural acute hemodynamic decompensation in patients undergoing catheter ablation of electrical storm. Heart Rhythm.

[28] Baratto F., Pappalardo F., Oloriz T., Bisceglia C., Vergara P., Silberbauer J., Albanese N., Cireddu M., D’Angelo G., Di Prima A.L. (2016). Extracorporeal Membrane Oxygenation for He-modynamic Support of Ventricular Tachycardia Ablation. Circ. Arrhythmia Electrophysiol..

[29] Kusa S., Miller M.A., Whang W., Enomoto Y., Panizo J.G., Iwasawa J., Choudry S., Pinney S., Gomes A., Langan N. (2017). Outcomes of Ventricular Tachycardia Ablation Using Percutaneous Left Ventricular Assist Devices. Circ. Arrhythmia Electrophysiol..

[30] Jared Bunch T., Mahapatra S., Madhu Reddy Y., Lakkireddy D. (2012). The role of percutaneous left ventricular assist devices during ventricular tachycardia ablation. Europace.

[31] Mariani S., Napp L.C., Kraaier K., Li T., Bounader K., Hanke J.S., Dogan G., Schmitto J.D., Lorusso R. (2021). Prophylactic mechanical circulatory support for protected ventricular tachycardia ablation: A meta-analysis of the literature. Artif. Organs.

[32] Aryana A., O’neill P.G., Gregory D., Scotti D., Bailey S., Brunton S., Chang M., D’avila A. (2014). Procedural and clinical outcomes after catheter ablation of unstable ventricular tachycardia supported by a percutaneous left ventricular assist device. Heart Rhythm.

[33] Aryana A., D'Avila A., Cool C.L., Miller M.A., Garcia F.C., Supple G.E., Dukkipati S.R., Lakkireddy D., Bunch T.J., Bowers M.R. (2017). Outcomes of catheter ablation of ventricular tachycardia with mechanical hemodynamic support: An analysis of the Medicare database. J. Cardiovasc. Electrophysiol..

[34] Kuo L., Muser D., Shirai Y., Lin A., Liang J., Schaller R.D., Hyman M., Kumareswaran R., Arkles J., Supple G.E. (2020). Periprocedural Acute Kidney Injury in Patients with Structural Heart Disease Undergoing Catheter Ablation of VT. JACC Clin. Electrophysiol..

[35] Carbucicchio C., Della Bella P., Fassini G., Trevisi N., Riva S., Giraldi F., Baratto F., Marenzi G., Sisillo E., Bartorelli A. (2009). Percutaneous Cardiopulmonary Support for Catheter Ablation of Unstable Ventricular Arrhythmias in High-Risk Patients. Herz.

[36] Abuissa H., Roshan J., Lim B., Asirvatham S.J. (2010). Use of the impellaTM microaxial blood pump for ablation of hemodynamically un-stable ventricular tachycardia. J. Cardiovasc. Electrophysiol..

[37] Miller M.A., Dukkipati S.R., Mittnacht A.J., Chinitz J.S., Belliveau L., Koruth J.S., Gomes J.A., d’Avila A., Reddy V.Y. (2011). Activation and entrainment mapping of he-modynamically unstable ventricular tachycardia using a percutaneous left ventricular assist device. J. Am. Coll Cardiol..

[38] Lü F., Eckman P.M., Liao K.K., Apostolidou I., John R., Chen T., Das G.S., Francis G.S., Lei H., Trohman R.G. (2013). Catheter ablation of hemodynamically unstable ventricular tachycardia with mechanical circulatory support. Int. J. Cardiol..

[39] Reddy Y.M., Chinitz L., Mansour M., Bunch T.J., Mahapatra S., Swarup V., Di Biase L., Bommana S., Atkins D., Tung R. (2014). Percutaneous left ventricular assist devices in ven-tricular tachycardia ablation: Multicenter experience. Circ. Arrhythmia Electrophysiol..

[40] Mariani S., Napp L.C., Coco V.L., Delnoij T.S., Luermans J.G., ter Bekke R.M., Timmermans C., Li T., Dogan G., Schmitto J.D. (2020). Mechanical circulatory support for life-threatening arrhythmia: A systematic review. Int. J. Cardiol..

[41] Ostadal P., Mlcek M., Holy F., Horakova S., Kralovec S., Skoda J., Petru J., Kruger A., Hrachovina V., Svoboda T. (2012). Direct Comparison of Percutaneous Circulatory Support Systems in Specific Hemodynamic Conditions in a Porcine Model. Circ. Arrhythmia Electrophysiol..

[42] Jain A., Arora S., Patel V., Raval M., Modi K., Arora N., Desai R., Bozorgnia B., Bonita R. (2023). Etiologies and Predictors of 30-Day Readmission in Heart Failure: An Updated Analysis. Int. J. Heart Fail..

[43] Bharadwaj A., McCabe M.D., Contractor T., Ben Kim H., Sakr A., Hilliard A., Mandapati R., Bhardwaj R. (2022). Multidisciplinary Approach to Hemodynamic Management During High-Risk Ventricular Tachycardia Ablation. JACC Case Rep..

[44] Bella P.D., Baratto F., Tsiachris D., Trevisi N., Vergara P., Bisceglia C., Petracca F., Carbucicchio C., Benussi S., Maisano F. (2013). Management of ventricular tachycardia in the setting of a dedicated unit for the treatment of complex ventricular arrhythmias: Long-term outcome after ablation. Circulation.

[45] Pothineni N.V.K., Enriquez A., Kumareswaran R., Garcia F., Shah R., Wald J., Bermudez C., Muser D., Marchlinski F.E., Santangeli P. (2023). Outcomes of a PAINESD score-guided multidis-ciplinary management approach for patients with ventricular tachycardia storm and advanced heart failure: A pilot study. Heart Rhythm.

